# The Centromere: Chromatin Foundation for the Kinetochore Machinery

**DOI:** 10.1016/j.devcel.2014.08.016

**Published:** 2014-09-08

**Authors:** Tatsuo Fukagawa, William C. Earnshaw

**Affiliations:** 1 Department of Molecular Genetics, National Institute of Genetics and Graduate University for Advanced Studies (SOKENDAI), Mishima, Shizuoka 411-8540, Japan; 2 Wellcome Trust Centre for Cell Biology, University of Edinburgh, King’s Buildings, Mayfield Road, Edinburgh, EH9 3JR, UK

## Abstract

Since discovery of the centromere-specific histone H3 variant CENP-A, centromeres have come to be defined as chromatin structures that establish the assembly site for the complex kinetochore machinery. In most organisms, centromere activity is defined epigenetically, rather than by specific DNA sequences. In this review, we describe selected classic work and recent progress in studies of centromeric chromatin with a focus on vertebrates. We consider possible roles for repetitive DNA sequences found at most centromeres, chromatin factors and modifications that assemble and activate CENP-A chromatin for kinetochore assembly, plus the use of artificial chromosomes and kinetochores to study centromere function.

## Main Text

### Introduction

In the 1930s, the site where chromosomes associate with the spindle during cell division was independently given two names: the “centromere” (Darlington, 1936) and the “kinetochore” (Schrader, 1939). The terms were long thought redundant, but it has recently proven useful to differentiate between them.

Centromeres, classically defined in genetics as regions of suppressed meiotic recombination (Beadle, 1932), were later recognized as the primary constriction of mitotic chromosomes. Centromeres are enriched in satellite repeats (Pardue and Gall, 1970) that stain dark by C-banding (McKay, 1973). When electron microscopy revealed a multilayered structure that binds to microtubules at the surface of centromeres (Luykx, 1965, Brinkley and Stubblefield, 1966, Jokelainen, 1967), that structure was termed the “kinetochore.” The centromere is now generally accepted to be a chromatin structure that specifies where the kinetochore will form. Kinetochores are complex protein structures lacking DNA (Cooke et al., 1993).

Molecular studies of the centromere/kinetochore began with the discovery that some patients with scleroderma spectrum disease (e.g., CREST syndrome) have anti-centromere autoantibodies (ACA) (Moroi et al., 1980). Three antigens, CENP-A, CENP-B, and CENP-C, are recognized by those sera (Earnshaw and Rothfield, 1985). CENP-A is a centromere-specific histone H3 variant (Palmer et al., 1987, Earnshaw et al., 2013). CENP-A and CENP-C localize to the inner kinetochore (Saitoh et al., 1992, Vafa and Sullivan, 1997, Warburton et al., 1997). CENP-B is an α-satellite (human centromeric DNA)-binding protein (Earnshaw et al., 1987). To date, over 100 kinetochore components have been identified. Aspects of kinetochore function that are beginning to be well understood include microtubule binding, chromosome movement, and checkpoint signaling. (For reviews, see Rieder, 1982, Maiato et al., 2004, Cheeseman and Desai, 2008, Santaguida and Musacchio, 2009, Perpelescu and Fukagawa, 2011, Vleugel et al., 2012).

The organization and functions of centromeric chromatin remain less understood subjects of active study. In this review, we discuss recent progress in understanding the organization, composition, and assembly of centromeric chromatin and DNA.

### Point and Regional Centromeres of Model Organisms

Budding yeast *Saccharomyces cerevisiae* centromeres occupy a ∼125 bp DNA sequence (Hegemann and Fleig, 1993, Clarke, 1998), now termed a “point centromere” (Pluta et al., 1995). They include three conserved DNA elements (CDE), CDEI, CDEII, and CDEIII that form a single nucleosome containing CENP-A (Cse4 in *S. cerevisiae*) (Stoler et al., 1995). The CDEI-III sequences are necessary and sufficient for active centromere formation, and a single base mutation in CDEIII can abolish centromere function (Clarke, 1998).

Centromeres of the fission yeast *Schizosaccharomyces pombe* encompass 40–100 kb containing a central core (cnt) of 4–7 kb, where the kinetochore forms, flanked by repeated sequences (otr) that form heterochromatin (Takahashi et al., 1992). This configuration is known as a “regional centromere” (Pluta et al., 1995). Both cnt and otr sequences are required for centromere function (Baum et al., 1994). Heterochromatin is required during de novo centromere formation (Folco et al., 2008). Transcription of otr and subsequent transcript processing by the RNAi machinery direct formation of pericentromeric heterochromatin (Volpe et al., 2002, Chen et al., 2008).

Regional centromeres of the pathogenic yeast *Candida albicans* map to unique sequences of ∼3 kb on each of the 8 chromosomes (Sanyal et al., 2004). Although a truncated minichromosome containing a fragment from the centromere retained centromere activity, centromeric activity was not reconstituted when naked minichromosome DNA was introduced back into yeast (Baum et al., 2006). It was suggested that an unusual chromatin structure detected at endogenous centromeres might be involved in epigenetic specification of the centromere in this yeast.

The regional centromere is the most common organization for centromeres in humans and most model organisms characterized to date, including *Neurospora crassa* (Centola and Carbon, 1994), *Arabidopsis thaliana* (Copenhaver et al., 1999), *Drosophila melanogaster* (Sun et al., 2003), and *Oryza sativa* (rice) (Nagaki et al., 2004). Common features of regional centromeres include the lack of a “magic” DNA sequence, the presence of complex repeated DNAs (satellite repeats together with centromeric retrotransposons), and an involvement of some sort of epigenetic mechanism in specification of the site for kinetochore assembly.

Nematodes, some insects, and plants assemble a “holocentromere” (originally, a holokinetochore) that extends along the entire length of the chromosome (Hughes-Schrader and Ris, 1941). Recent chromatin immunoprecipitation-on-chip (ChIP-chip) analyses in *Caenorhabditis elegans* (Gassmann et al., 2012) revealed that CENP-A occupies nonrepeated regions of 10–12 kb dispersed across about half of the genome and is excluded from loci that are transcribed in the germline and early embryo (for a contrasting view, see Steiner and Henikoff, 2014). Holocentromeres may consist of numerous CENP-A “seeds” dispersed along the chromosome that somehow cooperate to direct assembly of a functional kinetochore.

### Kinetochores Assemble on Repetitive DNA Sequences in Most Organisms

**Figure 1:**
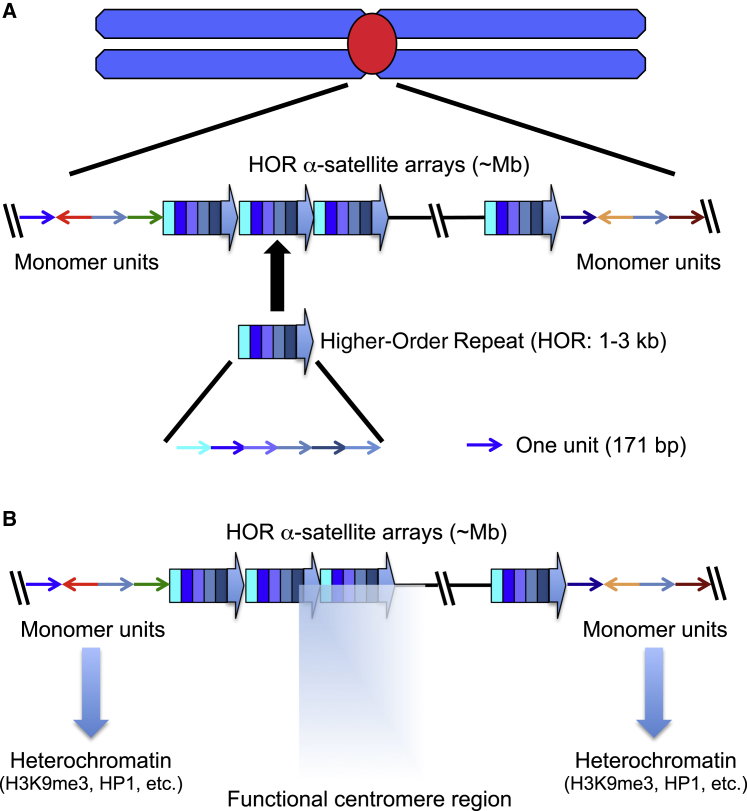
Genomic Organization of Human Centromeres (A) Human centromeres contain α-satellite sequences. In the inner core of the centromere, α-satellite monomers 171 bp long are organized into higher-order repeat (HOR) units, which are amplified, spanning 0.3–5 Mb. Unordered monomer units flank the HOR region. (B) Functional centromeres form on a portion of the HOR, which might be newly evolved and homogenized. Disordered monomer unit sequences in pericentromeres are derived from an ancestral primate centromere. Pericentromere regions are highly heterochromatinized.

In situ hybridization first revealed that satellite repeats were located at centromeres of mouse chromosomes (Pardue and Gall, 1970). Subsequent studies identified human centromeric DNA as α-satellite (Manuelidis, 1978). This DNA, with its 171 bp consensus sequence (Vissel and Choo, 1987), exhibits a complex higher-order repeat (HOR) pattern, in which adjacent monomers may share no more than 50% sequence identity, but corresponding monomers among the HORs share >90% identity (Waye and Willard, 1989, Aldrup-Macdonald and Sullivan, 2014). Different-sized HORs are observed on differing human chromosomes, where they typically create a chromosome-specific array that spans 0.3–5 Mb within the centromere (Figure 1).

Sequence assembly across α-satellite arrays at human centromeres is difficult due to high sequence similarities within the arrays and variability between individuals. To date, complete maps across the HOR and into pericentromeric heterochromatin are available only for chromosomes 8, X, and Y (Schueler et al., 2001, Nusbaum et al., 2006, Miga et al., 2014). On the X, a repeating HOR structure is flanked by divergent α-satellite monomers not ordered into HOR (Figure 1) (Schueler et al., 2001). The functional kinetochore assembles on the HOR core, with monomeric α-satellite sequences comprising pericentromeric heterochromatin (Schueler et al., 2001).

The conservation of α-satellite sequences at all natural human centromeres suggested that these HORs are required for centromere identity or function (Schueler et al., 2001). However, this was ruled out by the discovery of human dicentric chromosomes containing α-satellite DNA arrays that do not nucleate kinetochore formation (Earnshaw and Migeon, 1985, Earnshaw et al., 1989) and functional neocentromeres lacking α-satellite sequences (Voullaire et al., 1993).

Thus, although there are exceptions (see below), centromere regions from most organisms contain repetitive sequences, suggesting that those sequences contribute to important aspects of centromere function.

### Repetitive Sequences in Centromeres May Allow Kinetochore Plasticity

If kinetochores can form on nonrepetitive sequences, why do most centromeres contain repetitive sequences? One possibility is that repetitive sequences direct the formation of pericentromeric heterochromatin (Ekwall, 2007), with its molecular signature of heterochromatin protein 1 (HP1) bound to histone H3 trimethylated on lysine 9 (H3K9me3) (Minc et al., 1999, Nakayama et al., 2001, Yamagishi et al., 2008) (Figure 1). Although neocentromeres assembled on nonrepetitive DNA lack heterochromatin and yet function perfectly well in mitosis and meiosis (Alonso et al., 2010, Shang et al., 2013), pericentromeric heterochromatin, which consists of unordered monomer units of α-satellite in human, appears to provide a boundary between the kinetochore and flanking euchromatin regions in natural centromeres and might act as a barrier to centromere migration (Figure 1).

Pericentromeric heterochromatin recruits cohesin (Nonaka et al., 2002, Yamagishi et al., 2008, Gartenberg, 2009), and centromeric cohesion is particularly important in meiosis I where sister chromatids must remain paired. A requirement for strong centromeric cohesion in meiosis I could select for the accumulation of repetitive sequences in centromeres over evolutionary time scales.

In humans, the CENP-A associated domain at natural centromeres varies between 200 and 2,000 kb on different chromosomes and individuals (Sullivan et al., 2011). Thus, kinetochores form only over a portion of the α-satellite arrays (Figure 1). Due to the repetitive nature of the underlying DNA at natural centromeres, the kinetochore region cannot be mapped unambiguously by modern ChIP methods. This can be done at human neocentromeres, where the CENP-A domain spans just 80–100 kb (Alonso et al., 2010, Hasson et al., 2013). Thus, neocentromeres are much smaller than native centromeres.

**Figure 2:**
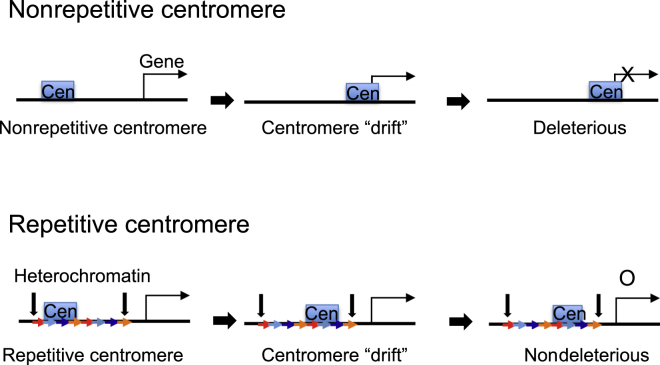
Proposed Role of Repetitive Sequences in Centromeres We postulate that the kinetochore location might be able to “drift” on the DNA. If a kinetochore moved over a gene, then it would suppress its expression. This would be deleterious for essential genes. At nonrepetitive centromeres, the probability of this drift affecting a flanking gene is higher than at repetitive centromeres, where the probability of affecting a gene is relatively low. We suggest that repetitive sequences might provide a safety area for centromere “drift.” Pericentromere regions at edge of centromeres might also function as a heterochromatin barrier between centromere and euchromatin regions.

Although active genes are present within rice centromeres (Nagaki et al., 2004), kinetochore formation across a transcribed gene strongly suppressed transcription of that gene on the chicken Z chromosome (Shang et al., 2013). Thus, kinetochore formation on essential genes would be expected to be deleterious. Because regional centromere position is not strictly specified by DNA sequence, it is possible that the kinetochore position on the underlying DNA might drift slightly (Figure 2). In this case, repetitive arrays could provide a safety buffer within which such drift would be harmless (Figure 2). It will be interesting in future studies of unique sequence centromeres to test whether kinetochore position is fixed or plastic.

### Neocentromeres Reveal Insights into Centromere Specification

Very rarely, disruption or inactivation of a natural centromere is followed by formation of a neocentromere at a new locus on a chromosome arm. Over 100 neocentromeres have been described in human clinical samples (Marshall et al., 2008). They form on diverse DNA sequences and are not associated with α-satellite arrays, thus providing strong evidence that human centromeres are specified by sequence-independent epigenetic mechanisms.

**Figure 3:**
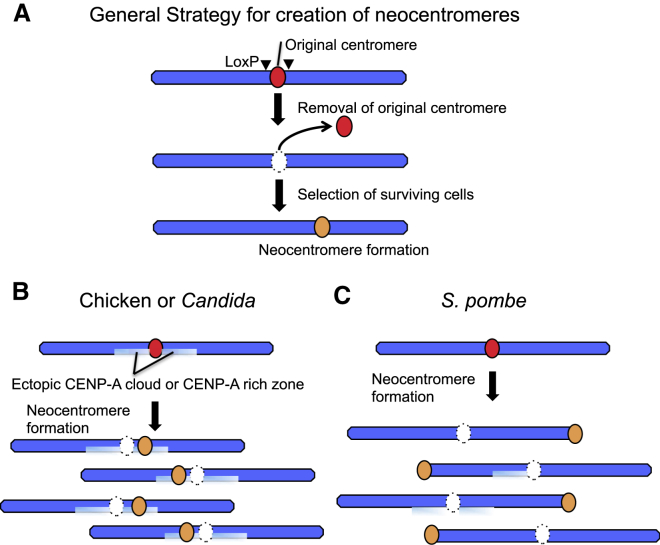
Neocentromere Generation in Various Experimental Systems (A) Isolation of surviving cells after removal of the endogenous centromere on a chromosome by homologous recombination between two LoxP loci flanking the centromere. Surviving cells usually have segregation of the chromosome directed by a neocentromere. (B) Ectopic CENP-A is assembled into a “CENP-A cloud” flanking the endogenous centromere in chicken cells. A CENP-A-rich region might be created by specific 3D packaging of the genome in *Candida* nuclei. The ectopic CENP-A functions as a site of preferential neocentromere formation in chicken or *Candida* cells when the endogenous centromere is disrupted. In contrast, neocentromeres are preferentially formed on telomeres in *S. pombe*.

Experimental generation of neocentromeres in model organisms has yielded insights into the process of neocentromere formation. In *D. melanogaster*, neocentromeres obtained following γ-irradiation-based chromosome breakage formed near the pericentromeric region of the X chromosome (Murphy and Karpen, 1995). Subsequently, neocentromeres were generated in *S. pombe* (Ishii et al., 2008) and *C. albicans* (Ketel et al., 2009) following targeted deletion of the original centromere and genetic selection for retention of the chromosome (Figure 3). *C. albicans* neocentromeres formed either in transcriptionally active or intergenic regions near the natural centromeres (Ketel et al., 2009, Thakur and Sanyal, 2013). In contrast, *S. pombe* neocentromeres preferentially formed near telomeric heterochromatin and efficient neocentromere formation required heterochromatin proteins (Ishii et al., 2008).

Recently, chromosome engineering has allowed the efficient isolation of neocentromeres in chicken DT40 cells. Conditional deletion of the centromeres of chromosome Z or 5 led to neocentromere formation at a frequency of ∼3 × 10^−6^ on a wide range of both transcriptionally active and inactive sequences (Figure 3) (Shang et al., 2013). Thus, neocentromeres can be “seeded” on either transcriptionally active or inactive regions of the genome, and heterochromatin is not required for centromere formation. However, neocentromere formation caused a significant drop in gene transcription when a neocentromere formed on an actively transcribed gene on the Z chromosome.

Chicken neocentromeres have a remarkably constant size of ∼40 ± 6 kb, and CENP-A domains did not expand even in cells overexpressing CENP-A (Shang et al., 2013). In contrast, increased levels of CENP-A expression human cells resulted in increased CENP-A incorporation at centromeres (Bodor et al., 2014). CENP-A levels are regulated by a ubiquitin-dependent pathway in yeasts (Ranjitkar et al., 2010, Kitagawa et al., 2014), and the mechanisms governing centromere size may vary.

*Drosophila*, barley, *Candida*, and chicken neocentromeres all formed close to the natural centromeres (Figure 3) (Williams et al., 1998, Nasuda et al., 2005, Shang et al., 2013, Thakur and Sanyal, 2013). Possibly, patterns of neocentromere formation are determined by noncentromeric incorporation of CENP-A (Figure 3) in regions with high histone turnover (Lacoste et al., 2014). Remarkably, significant levels of CENP-A in vertebrates are incorporated into chromatin at noncentromeric sites (30% in chicken and 74% in human RPE1 cells [Shang et al., 2013, Bodor et al., 2014]). Importantly, given the size of human genome, this still corresponds to a ∼50× concentration increase at centromeres (Bodor et al., 2014). In chicken, the ectopic CENP-A is enriched in chromatin flanking the natural centromeres (the “CENP-A cloud”). Noncentromeric Cse4 (CENP-A) is also observed in budding yeast (Camahort et al., 2009, Lefrançois et al., 2009, Lefrançois et al., 2013) and might form a CENP-A “cloud” (Kerry Bloom, personal communication). The “cloud” was not detected in *Candida* (Thakur and Sanyal, 2013), where it was proposed that neocentromeres might form in a CENP-A-rich zone created by specific three-dimensional packaging of the genome in nuclei.

We suggest that the ectopic “CENP-A cloud” functions to seed neocentromere formation when natural centromeres are disrupted (Figure 3). How centromere formation is suppressed in the ectopic “CENP-A cloud” and how this suppression is lifted once natural centromeres are compromised remain interesting questions for further study.

### Nonrepetitive Centromeres Found in Horses, Orangutans, and Chickens

Sequencing the horse genome revealed that the centromere of chromosome 11 lacked repetitive sequences (Wade et al., 2009). The CENP-A domain of horse centromere 11 is ∼90 kb (Wade et al., 2009), similar to a typical human neocentromere (80–100 kb) (Alonso et al., 2010). Chickens also have nonrepetitive centromeres on chromosomes 5, 27, and Z (the others are repetitive) (Shang et al., 2010). These nonrepetitive centromeres are only ∼40kb long based on CENP-A ChIP-seq analysis (Shang et al., 2010). Interestingly, orangutans also have one nonrepetitive centromere (Locke et al., 2011).

These nonrepeated centromeres might be evolutionally new centromeres (ENCs) that formed initially as neocentromeres, then become fixed in the population. It appears that over time, ENCs acquire repetitive DNA elements—presumably to stabilize them as proposed above.

### Human Artificial Chromosomes and Epigenetic Engineering of Centromeric Chromatin

Given the powerful insights obtained by formation of artificial chromosomes in yeasts (Murray and Szostak, 1983), it was thought that formation of artificial chromosomes in human cells might lead to important insights into centromere structure and function. The first human artificial chromosomes (HACs, also known as MACs or mammalian artificial chromosomes) were formed in cells transfected with a DNA cocktail including an α-satellite array, genomic DNA, and telomeric sequences (Harrington et al., 1997). Subsequently, it was reported that yeast artificial chromosomes containing α-satellite DNA and retrofitted with telomeres could also form HACs (Ikeno et al., 1998). Circular BACs lacking telomeric sequences also give rise to stable HACs and are easier to construct (Ebersole et al., 2000).

A number of general principles emerged from these studies. Only α-satellite DNA with a regular HOR repeat structure and with CENP-B binding sites is functional for HAC formation (Ohzeki et al., 2002). This was paradoxical as CENP-B knockout mice are viable and fertile (Hudson et al., 1998, Kapoor et al., 1998, Perez-Castro et al., 1998). Subsequent studies revealed that CENP-B appears to manage heterochromatin formation during de novo centromere formation (Okada et al., 2007), and it also appears to be required for efficient recruitment of CENP-C to kinetochores (Daniele Fachinetti and Don Cleveland, personal communication). Interestingly, either excessive heterochromatin or excessive transcription flanking the α-satellite DNA sequences interferes with de novo centromere formation (Nakashima et al., 2005, Ohzeki et al., 2012).

HACs have thus far proved to be of only limited utility in understanding the mechanisms of human centromere formation. HACs are observed only after multiple generations in cell culture during which an unknown sequence of events has occurred. HACs identified to date are considerably larger than the input DNA, and physical characterization of one HAC revealed that it had undergone a complex set of rearrangements and acquired sequences from the arm of chromosome 13 (Kouprina et al., 2013).

**Figure 4:**
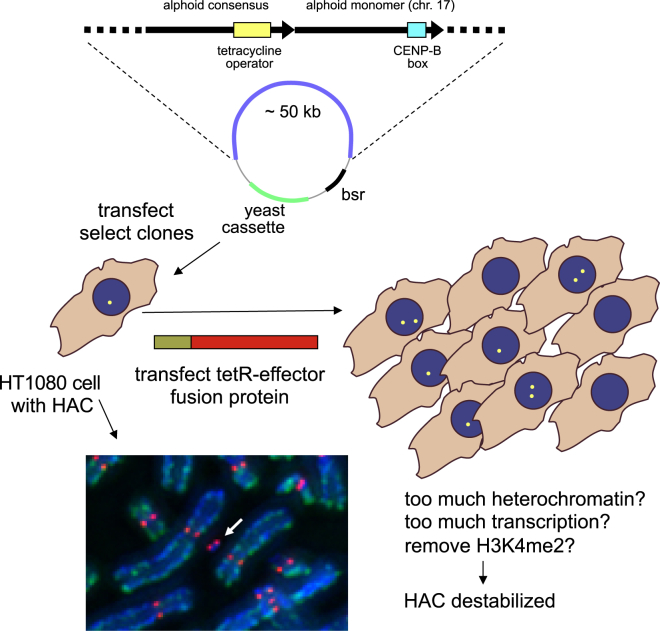
Use of Human Artificial Chromosomes to Study Centromeric Chromatin A synthetic α-satellite DNA construct was used to generate human artificial chromosomes (HACs) in HT1080 fibrosarcoma cells. An example of the HAC (white arrow) stained for kinetochore protein CENP-C (red) and DNA (blue) is shown. The presence of tetracycline operator sequences in the synthetic array allows targeting of chimeric proteins containing a range of chromatin modification activities into the HAC centromere. These studies reveal that centromere activity requires finely balanced transcription in a repressive environment. Micrograph by Jan Bergmann.

Despite these limitations, the alphoid^tetO^ HAC has recently begun to yield insights about the chromatin environment required within centromeric DNA in and around the kinetochore (Nakano et al., 2008) (Figure 4). When tetracycline repressor fusion proteins were used to direct various chromatin modifiers into the centromeric chromatin, this revealed that excessive heterochromatin or excessive transcriptional activity within the centromere are incompatible with kinetochore assembly and propagation (Nakano et al., 2008, Cardinale et al., 2009, Bergmann et al., 2012). Interestingly, a moderate (10×) activation of transcription within the centromere is tolerated (Bergmann et al., 2012) (Figure 4). Consistent with these results, transcription and RNA polymerase have been detected in centromeric sequences, even in mitotic cells (Bergmann et al., 2011, Chan et al., 2012). It thus appears that kinetochore stability at human centromeres requires finely balanced RNA transcription within an otherwise silent chromatin environment.

### Are CENP-A-Containing Nucleosomes Different from Bulk Nucleosomes?

Centromeric chromatin forms a specialized structure in budding (Bloom and Carbon, 1982) and fission yeasts (Takahashi et al., 1992). CENP-A sequences from model organisms have highly variable N-terminal tails (Black and Cleveland, 2011), but in human cells, centromere targeting is directed by the 22 amino acid (aa) CENP-A targeting domain (CATD) located in the histone-fold region (Black et al., 2004). The CATD binds the CENP-A-specific chaperone HJURP (Dunleavy et al., 2009, Foltz et al., 2009). The CENP-A nucleosome core is rigid (Black et al., 2004), but overall the DNA wraps less tightly in both natural and neocentromeres than in conventional nucleosomes (Hasson et al., 2013). These differences suggest that CENP-A nucleosomes are distinct from canonical H3 nucleosomes. Indeed, conflicting models have been proposed for the structure of CENP-A-containing nucleosomes (Black and Cleveland, 2011), and a spirited ongoing controversy concerns whether they are octameric or tetrameric.

Octameric CENP-A nucleosomes can be reconstituted with recombinant histones (Yoda et al., 2000, Sekulic et al., 2010) and CENP-A-containing nucleosomes purified from human cells contained stoichiometric CENP-A, H4, H2A, and H2B, with two CENP-A molecules per nucleosome (Shelby et al., 1997, Foltz et al., 2006). X-ray crystallography revealed that the structure of reconstituted CENP-A nucleosomes resembles canonical nucleosomes with subtle differences (Tachiwana et al., 2011).

In contrast, Henikoff and Dalal argued that CENP-A nucleosomes form a tetrameric hemisome containing a single copy of CENP-A, H4, H2A, and H2B in *Drosophila* cells (Dalal et al., 2007). They also proposed that DNA wraps around CENP-A hemisomes with a handedness opposite to that found in canonical nucleosomes (Furuyama and Henikoff, 2009). Using atomic force microscopy (AFM), they suggested that CENP-A nucleosomes are half the height of canonical nucleosomes (Dimitriadis et al., 2010). More recently, studies in human cells (Bui et al., 2012) and budding yeast (Shivaraju et al., 2012) proposed that the CENP-A-containing nucleosomes are dynamic, oscillating between octameric and tetrameric forms during cell-cycle progression.

Although this debate is still active, recent crosslinking (Zhang et al., 2012), photobleaching (Padeganeh et al., 2013), and AFM experiments (Miell et al., 2013) suggest that most CENP-A-containing nucleosomes are octameric, with a more rigid core than canonical nucleosomes (Black et al., 2007). A recent study using fully functional Cse4 with an internal tdEos tag after Leu81 in the N-terminal region showed clearly that budding yeast kinetochores have two copies of Cse4 in a single nucleosome (Wisniewski et al., 2014). The authors also demonstrated that studies in which Cse4 copy number appeared to vary across the cell cycle can be explained by delays in the activation of fluorescent proteins.

This controversy appears to have a life of its own, and studies using isolated CENP-A nucleosomes could be confounded by the fact that many CENP-A nucleosomes are noncentromeric. However, although there is still no absolute consensus, most emerging data appear to support the existence of octameric CENP-A nucleosomes in vivo.

Even if CENP-A-containing nucleosomes are octameric, some of these could be heterotypic, with both CENP-A and H3. CENP-A/H3.3 heterotypic nucleosomes can indeed form following CENP-A overexpression, but this is mostly in noncentromere regions (Lacoste et al., 2014). Homotypic CENP-A/CENP-A nucleosomes predominate at centromeres (Shelby et al., 1997, Hori et al., 2014, Lacoste et al., 2014).

### How Are CENP-A Nucleosomes Distributed in Centromeres?

**Figure 5:**
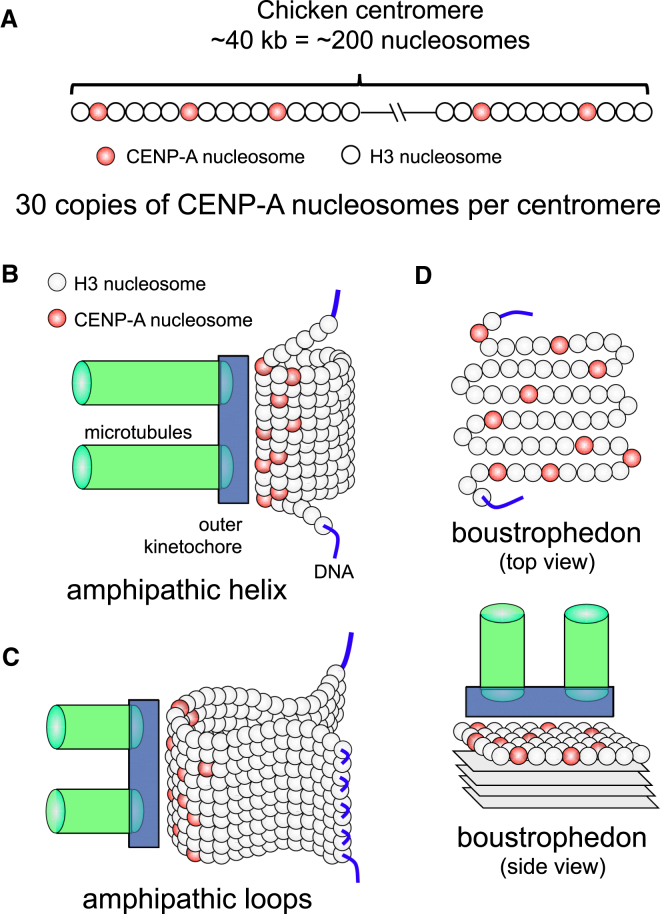
Organization of CENP-A and H3 Nucleosomes in Centromeres (A) Based on ChIP-seq analysis centromeres are ∼40 kb long in chicken, corresponding to 200 nucleosomes per centromere. Of these, 30 are predicted to contain CENP-A (roughly 1 in 6–8 centromeric nucleosomes). Thus, centromeric chromatin is largely composed of nucleosomes containing histone H3. (B and C) The CENP-A chromatin was originally suggested to form an amphipathic organization, with CENP-A on the exterior facing the kinetochore, and H3 largely on the interior. This chromatin was proposed to form either a helix or loop structure. The diagram in (B and C) is based on Blower et al. (2002), but modified to show the lower occupancy of CENP-A nucleosomes in the centromeric chromatin. (D) The boustrophedon model of centromeric CENP-A-containing chromatin was proposed based on super-resolution microscopy (Ribeiro et al., 2010).

A critical question is how many CENP-A nucleosomes are required to define a kinetochore. Immunofluorescence on extended chromatin fibers (Blower et al., 2002), superresolution microscopy (Ribeiro et al., 2010), and biochemical analyses (Blower et al., 2002, Hori et al., 2008) all suggest that centromeres consist of islets of CENP-A nucleosomes interspersed between regions containing H3 nucleosomes (Figure 5). In *S. cerevisiae*, elegant biochemical and quantitative imaging experiments detected a single Cse4/CENP-A nucleosome in the point centromere (Furuyama and Biggins, 2007, Joglekar et al., 2008). Other microscopy experiments suggest that other budding yeast strains might have up to three CENP-A nucleosomes per centromere (Lawrimore et al., 2011). However, these are technically deceptively complex experiments (Wisniewski et al., 2014).

Using *S. cerevisiae* as a standard (assuming a single CENP-A nucleosome per centromere), *S. pombe* centromeres were estimated to have 3 CENP-A nucleosomes (Joglekar et al., 2008) and chicken DT40 cells to have 30 (Johnston et al., 2010). Because CENP-A associated regions are ∼5 kb (∼25 nucleosomes) in S. *pombe* and ∼40 kb (∼200 nucleosomes) in chicken, and assuming 200 bp per nucleosome, this suggests that one in six to eight centromeric nucleosomes contains CENP-A (Figure 5). Recent studies report larger numbers of CENP-A nucleosomes in *S. pombe* (Coffman et al., 2011, Lando et al., 2012). A typical human neocentromere of 80–100 kb (400–500 nucleosomes) should contain ∼100 CENP-A nucleosomes (200 copies of CENP-A) per centromere (Figure 5). This is in remarkable agreement with the recent conclusion that human RPE1 cells have ∼100 CENP-A nucleosomes per mitotic kinetochore (Bodor et al., 2014). Considering copy-number estimates for other kinetochore proteins and numbers of microtubules (10–20 per kinetochore), this value of 30–100 CENP-A nucleosomes per vertebrate kinetochore appears reasonable (Figure 5).

### Folding of the CENP-A Chromatin Fiber at Centromeres

Analysis of extended centromeric chromatin fibers revealed that CENP-A forms clusters that alternate with chromatin containing canonical histone H3 (Blower et al., 2002). The H3 nucleosomes carry the transcription-associated modification H3K4me2 and define a specialized class of chromatin termed “centrochromatin” (Sullivan and Karpen, 2004). It was proposed that CENP-A was packaged either into an amphipathic-like solenoidal superhelix or as radial loops, with CENP-A nucleosomes clustered on the outer surface of the chromatin and H3 internal (Blower et al., 2002) (Figure 5).

Two types of super-resolution microscopy combined to challenge this interpretation. High-resolution dual-label microscopy showed that CENP-T, an important linker between the chromatin and the outer kinetochore (Hori et al., 2008, Suzuki et al., 2011), was located significantly outside of CENP-A (Joglekar et al., 2009, Wan et al., 2009, Varma et al., 2013). PALM microscopy of unfolded fibers derived from chicken kinetochores found CENP-T in regions of H3 nucleosomes (Ribeiro et al., 2010). In the amphipathic helix-loop model, this would place CENP-T on the inside, in direct contradiction with the dual-label microscopy mapping measurements.

To account for the superresolution mapping data, it was suggested that CENP-A chromatin might be organized as a sinusoidally folded patch, or boustrophedon, at the surface of the centromeric chromatin (Ribeiro et al., 2010). The boustrophedon was proposed to be 4–5 layers deep (Figure 5), consistent with the 10 nm diameter of the nucleosome and the ∼60 nm thickness of the kinetochore plate typically observed in electron micrographs (Rieder, 1982). Thus, the architecture of CENP-A chromatin and the boustrophedon model remains questions in need of further experimentation.

### The Paradoxical Timing of CENP-A Incorporation into Centromeres

New CENP-A is incorporated into the centromere during early G1 phase in vertebrate cells, following the drop in mitosis-associated CDK activity (Jansen et al., 2007, Silva et al., 2012). This was unexpected, because most canonical H3 is incorporated into chromatin during DNA replication. Furthermore, it means that cells traverse mitosis with only half of their maximal complement of CENP-A. This is not what one might have predicted given that centromeres perform their most important functions during mitosis.

Budding yeast Cse4 (CENP-A) is incorporated into centromeres during S-phase coupled with DNA replication and following the complete removal of preexisting Cse4 (Pearson et al., 2004, Wisniewski et al., 2014). Thus CENP-A incorporation timing differs between yeast and vertebrates. In the latter, histone H3.3 incorporated into centromeres during S-phase might function as a placeholder for new CENP-A deposition in the next G1 (Dunleavy et al., 2011).

Genetic analysis in *S. pombe* identified Mis16 and Mis18 as factors involved in CENP-A localization (Hayashi et al., 2004). Mis16 is the *S. pombe* homolog of vertebrate pRab46/48, which is involved in histone H3 incorporation into chromatin. In vertebrates, the two isoforms Mis18α/β form a complex with M18BP1 (Fujita et al., 2007), also known as KNL-2 (Maddox et al., 2007). Depletion of pRab46/48 or Mis18α/β in human cells reduces CENP-A incorporation into centromeres (Hayashi et al., 2004, Fujita et al., 2007), suggesting a conserved role for these proteins.

**Figure 6:**
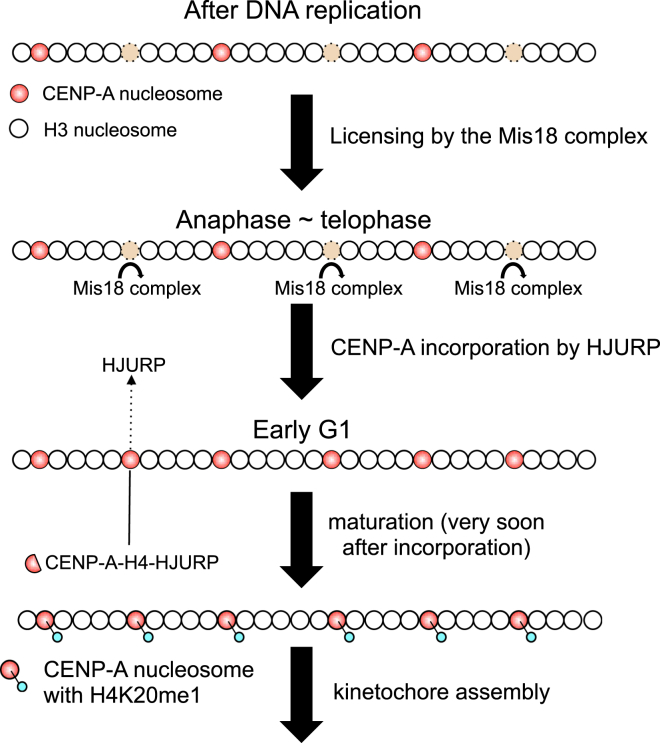
The Mis18 Complex Licenses Chromatin for CENP-A Incorporation During replication, CENP-A nucleosomes are divided between the two daughter DNA molecules and are either replaced with H3 (H3.1/H3.3) nucleosomes or leave nucleosome free gaps. During anaphase to telophase, the Mis18 complex localizes to centromeres. Soluble CENP-A-H4 binds to the chaperone HJURP before incorporation, and during early G1 the CENP-A-H4-HJUPRP complex is recruited into the licensed chromatin by binding to the Mis18 complex. Following CENP-A incorporation, H4K20 in CENP-A nucleosomes is monomethylated. This maturation step is required for certain of the constitutive centromere associated network (CCAN) proteins to assemble and subsequently direct assembly of a functional kinetochore.

The timing of CENP-A incorporation is regulated by Mis18α/β, which is recruited to centromeres at least in part via Mis18BP1 binding to CENP-C (Moree et al., 2011, Dambacher et al., 2012, McKinley and Cheeseman, 2014). Mis18α/β starts to localize to centromeres during anaphase and remains there during telophase. Its levels fall dramatically in G1 phase (Hayashi et al., 2004, Fujita et al., 2007). Mis18α/β thus appears to function as a licensing factor for CENP-A incorporation (Figure 6).

The Mis18 complex must be phosphorylated by Polo-like kinase 1 (Plk1) during G1 to facilitate CENP-A incorporation (McKinley and Cheeseman, 2014). Because CDK1 activity negatively regulates Mis18 complex-derived CENP-A incorporation (Silva et al., 2012), CENP-A incorporation might be controlled by a two-step regulatory mechanism governed by Plk1 and CDK1.

The Mis18 complex does not directly bind to soluble CENP-A. Instead a CENP-A-specific chaperone, HJURP, binds soluble CENP-A-H4 complex before its chromatin incorporation during G1 phase (Dunleavy et al., 2009, Foltz et al., 2009) (Figure 6). The yeast and *Drosophila* homologs of HJURP are apparently Scm3 (Mizuguchi et al., 2007, Pidoux et al., 2009, Sanchez-Pulido et al., 2009, Williams et al., 2009) and Cal1 (Erhardt et al., 2008), respectively. Scm3 is found in hexameric Cse4 nucleosomes (Mizuguchi et al., 2007), which appear to be assembly intermediates (Camahort et al., 2007). The mechanism of CENP-A incorporation is thus widely conserved. Current data suggest that the Mis18 complex licenses centromeric chromatin by binding and recruiting a complex of HJURP-CENP-A-H4 (Figure 6).

The timing of CENP-A incorporation is critical for kinetochore assembly and function. If CENP-A incorporation is artificially deregulated by constitutive targeting of Mis18α to centromeres (this causes constitutive insertion of CENP-A across the cell cycle), then mitotic kinetochore function is strongly disrupted (McKinley and Cheeseman, 2014). It is not known why unscheduled incorporation of CENP-A has such a strong disruptive effect.

### How Does CENP-A Chromatin Become Competent for Kinetochore Assembly?

With the exception of Trypanosomids (Akiyoshi and Gull, 2014), CENP-A containing chromatin is a near universal feature of centromere specification. Yet with few examples (Guse et al., 2011, Mendiburo et al., 2011), targeting of ectopic CENP-A is not sufficient to trigger centromere formation (Van Hooser et al., 2001, Gascoigne et al., 2011). Indeed, because most chromosomal CENP-A is incorporated at ectopic sites that lack centromere activity (Shang et al., 2013, Bodor et al., 2014), this begs the question of how cells distinguish centromeric CENP-A from ectopic CENP-A. It is possible that additional modifications might “license” CENP-A containing chromatin, making it competent for kinetochore assembly. A recent study revealed that histone H4 Lys20 monomethylation (H4K20me1) specifically occurs at centromeric CENP-A chromatin (Hori et al., 2014). Because reducing H4K20me1 levels at centromeres causes mislocalization of CENP-H and CENP-T, this modification might help render CENP-A chromatin competent for kinetochore assembly (Figure 6). Other histone modifications of CENP-A nucleosomes or of centromeric nucleosomes containing canonical H3 might also be involved in formation of functional centromeric chromatin, possibly by influencing interactions with other kinetochore proteins such as CENP-C or CENP-N, which bind to CENP-A nucleosomes concentrated in centromeres (Carroll et al., 2009, Guse et al., 2011, Kato et al., 2013).

Understanding the mechanisms by which CENP-A chromatin induces subsequent kinetochore assembly remains an important area for future studies.

### Centromeric Chromatin Contributes to Sister Chromatid Cohesion

In addition to serving as a mark for kinetochore assembly, centromeric chromatin also contributes to chromosome segregation by binding cohesin and the chromosomal passenger complex (CPC) of Aurora B kinase, INCENP, Survivin, and Borealin (Carmena et al., 2012).

Cohesin regulates the cohesion of sister chromatids so that kinetochores can orient to opposite spindle poles and chromosomes can segregate equally during mitosis (Nasmyth and Haering, 2009). Pericentromeric heterochromatin in *S. pombe* recruits cohesin (Nonaka et al., 2002), and the link between heterochromatin and cohesin is conserved in other organisms (Gartenberg, 2009). Cohesin is also concentrated in the pericentromere in budding yeast where there is no canonical heterochromatin (Kiburz et al., 2005). Cohesion at centromeres is protected by proteins of the shugoshin family (Watanabe, 2005), which have recently been observed to also function in recruitment of the CPC (Gutiérrez-Caballero et al., 2012).

Shugoshin (Sgo1) forms part of a two-part mechanism for recruiting the CPC to centromeric heterochromatin. First, the checkpoint kinase Bub1 phosphorylates histone H2A at threonine 120 (H2AT120ph) in inner centromeres. Sgo1 binds to this histone mark and then recruits the CPC via an interaction with Borealin (Kawashima et al., 2007). Second, Haspin kinase phosphorylation of histone H3 threonine 3 (H3T3ph). Survivin binding to this histone mark is required for CPC targeting to centromeres (Kelly et al., 2010, Wang et al., 2010, Yamagishi et al., 2010). At centromeres, the CPC regulates kinetochore-microtubule interactions by phosphorylation of several kinetochore proteins and regulates spindle checkpoint signaling to delay mitotic progression if there are incorrect kinetochore-microtubule attachments (Carmena et al., 2012).

### Histone-fold Proteins that Bridge between the Centromere and Kinetochore

CENP-A and H3 nucleosomes are not the only proteins that directly bind to centromeric DNA and contribute to the formation of centromere-specific chromatin. Experiments aimed at isolating unknown centromere proteins identified CENP-T, CENP-W, CENP-S, and CENP-X, all of which directly bind to centromeric DNA (Okada et al., 2006, Hori et al., 2008, Amano et al., 2009). These proteins have histone folds and make a tetrameric CENP-T-W-S-X complex (Nishino et al., 2012). Because this complex can induce supercoils into DNA and its DNA binding surface resembles that of canonical nucleosomes, it might form a nucleosome-like structure at centromeres (Nishino et al., 2012).

**Figure 7:**
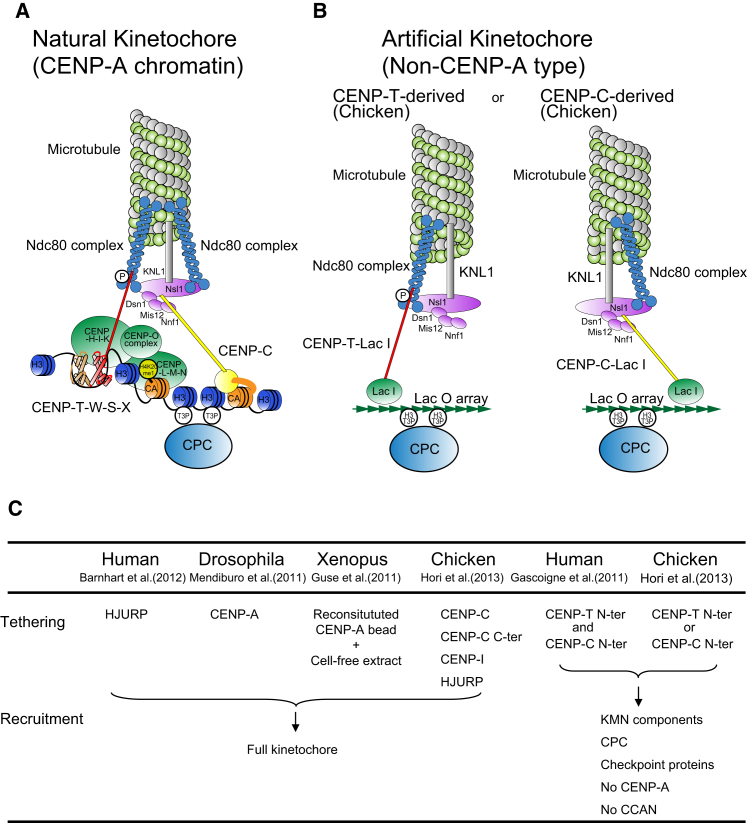
Molecular Architecture of Kinetochores (A) Molecular architecture of natural kinetochores. Centromere chromatin is crucial for centromere specification and kinetochore assembly at natural centromeres. At the base of the structure are CENP-A containing nucleosomes, centromere specific H3 nucleosomes, and a CENP-T-W-S-X nucleosome-like structure in centromere chromatin. Centromere-specific chromatin structure is established by coordination of these components. CCAN proteins assemble on the centromeric chromatin and the microtubule-binding complex is subsequently recruited to assemble the functional kinetochore. (B) When the CENP-T N terminus or CENP-C N terminus is tethered at a noncentromere locus using the LacI-LacO system, an artificial kinetochore forms on the noncentromeric LacO site. Most centromeric chromatin proteins, including CENP-A, are not detected in the artificial kinetochores. However, the chromosome passenger complex (CPC) and Ndc80 complex are recruited and the artificial kinetochores are fully functional. (C) Summary of studies on creation of artificial kinetochores. Tethering of CENP-T N terminus and/or CENP-C-N terminus can bypass the need for centromere-specific chromatin including CENP-A. CENP-A-mediated artificial kinetochores also have been created in human, chicken, and *Drosophila* cells and in Xenopus egg extracts.

Interestingly, the CENP-T-W-S-X complex induces positive supercoils into DNA (Takeuchi et al., 2014), whereas canonical histones induce negative supercoils. Analysis of yeast mini-chromosomes suggested that there are positive supercoils in centromere chromatin (Furuyama and Henikoff, 2009). Thus, in addition to CENP-A nucleosomes, the CENP-T-W-S-X nucleosome-like complex might contribute to formation of centromere-specific chromatin (Figure 7) (Takeuchi et al., 2014).

Although CENP-T-W-S-X tetramers are critical for centromere function, chromosome segregation still occurs in CENP-S- or CENP-X-deficient cells (Amano et al., 2009). This suggests that CENP-T-W and CENP-S-X can function independently. Indeed, CENP-S-X binds FANCM proteins at DNA damage sites (Singh et al., 2010, Yan et al., 2010). Further studies are needed to clarify how CENP-T-W, CENP-S-X, and CENP-T-W-S-X bind to DNA and how the tetramer contributes to formation of centromeric chromatin.

### Artificial Kinetochores Bypass the Need for Centromere-Specific Chromatin

One key function of the centromere is to regulate chromosomal interactions with microtubules. This interaction requires the Ndc80 complex in the kinetochore (Cheeseman et al., 2006, DeLuca et al., 2006). This suggested that if the Ndc80 complex could be artificially localized to an ectopic chromosomal locus, then that locus might function as an artificial kinetochore and direct chromosome segregation.

The CENP-T N-terminal region directly binds to the Ndc80 complex (Gascoigne et al., 2011, Nishino et al., 2013), while its C terminus contacts the centromeric DNA (Nishino et al., 2012). Remarkably, tethering the CENP-T N terminus at a noncentromeric locus using the LacI-LacO system resulted in creation of an artificial kinetochore that efficiently directed chromosome segregation following deletion of the natural centromere of the corresponding chromosome in chicken DT40 cells (Gascoigne et al., 2011, Hori et al., 2013). A second artificial kinetochore was also constructed by similar tethering of CENP-C to a Lac operator array (Hori et al., 2013). CENP-C recruits the Mis12 complex, which binds the Ndc80 complex (Gascoigne et al., 2011, Przewloka et al., 2011, Screpanti et al., 2011; Figure 7). Surprisingly, many centromere proteins, including CENP-A, were not detected in either artificial kinetochore (Hori et al., 2013). Together, these experiments reveal that recruiting the Ndc80 complex to centromeres is a major function of centromere chromatin. Thus, artificial recruitment of the complex can bypass the need for centromere-specific chromatin structure during kinetochore formation.

CENP-A-mediated artificial kinetochores also have been created in human, chicken, and *Drosophila* cells (Figure 7) (Barnhart et al., 2011, Mendiburo et al., 2011, Hori et al., 2013). In another approach, several aspects of kinetochore function were reconstituted in vitro on CENP-A nucleosome-coated beads using *Xenopus* egg extracts (Guse et al., 2011).

Artificial kinetochores are promising tools for kinetochore studies and genetic engineering. It will therefore be important to determine the efficiency with which they are able to direct chromosome segregation in animals and their ability to cope with attachment errors, which are a natural hazard of mitotic chromosome segregation.

### Perspectives

Even though centromeric chromatin can form both on specialized (often repetitive) DNA sequences and on other sequences that are not normally centromeric, its structure and composition are distinct from that of other chromatin regions. Centromeric chromatin consists of a relatively small number of CENP-A-containing nucleosomes distributed among centromere-specific H3 nucleosomes together with additional specialized DNA-binding proteins including the CENP-T-W-S-X complex (Figure 7). The centromere-specific chromatin structure is established by coordination of these factors with modification of the CENP-A nucleosomes, and together this lays the essential foundation for functional kinetochore assembly. Throughout this review, we have stressed that kinetochore function is distinct from centromeric chromatin. However, because kinetochores assemble on the surface of centromeres, it can be difficult to separate the functions of the two. By bypassing centromere function, artificial kinetochores might provide an excellent tool to tackle this issue.
